# Real-time Estimates in Early Detection of SARS

**DOI:** 10.3201/eid1201.050593

**Published:** 2006-01

**Authors:** Simon Cauchemez, Pierre-Yves Boëlle, Christl A. Donnelly, Neil M Ferguson, Guy Thomas, Gabriel M. Leung, Anthony J Hedley, Roy M. Anderson, Alain-Jacques Valleron

**Affiliations:** * Institut National de la Santé et de la Recherche Médicale, Paris, France;; † Université Pierre et Marie Curie, Paris, France;; ‡ Assistance Publique–Hôpitaux de Paris, Paris, France;; § Imperial College, London, United Kingdom;; ¶ University of Hong Kong, Hong Kong Special Administrative Region, People's Republic of China

**Keywords:** Severe acute respiratory syndrome, communicable diseases, emerging, disease outbreaks, epidemiologic methods, population surveillance, reproduction number, Hong Kong, research

## Abstract

We propose a Bayesian statistical framework for estimating the reproduction number *R* early in an epidemic. This method allows for the yet-unrecorded secondary cases if the estimate is obtained before the epidemic has ended. We applied our approach to the severe acute respiratory syndrome (SARS) epidemic that started in February 2003 in Hong Kong. Temporal patterns of *R* estimated after 5, 10, and 20 days were similar. Ninety-five percent credible intervals narrowed when more data were available but stabilized after 10 days. Using simulation studies of SARS-like outbreaks, we have shown that the method may be used for early monitoring of the effect of control measures.

## Abstract 2

A statistical method can be used for early monitoring of the effect of disease control measures.

The reproduction number *R* of an epidemic (the mean number of secondary cases infected by a single infectious case) is a key parameter for the analysis of infectious diseases because it summarizes the potential transmissibility of the disease and indicates whether an epidemic is under control (*R*<1). Up to now, this parameter has only been estimated retrospectively for periods from which all secondary cases had been detected. In terms of policy development and evaluation during the epidemic, obtaining estimates of the temporal trends in the reproduction number relating to as recent a time as possible would be critical.

If all incident cases could be traced to their index cases, estimating the reproduction number would simply be a matter of counting secondary cases. However, if tracing information is incomplete or ambiguous, modeling or statistical approaches are required. For example, a mathematical model for disease transmission fitted to available data can provide estimates of *R* (*1*). An approach requiring fewer assumptions has been proposed by Wallinga and Teunis (*2*), in which the distribution of the generation interval of the disease and the epidemic curve are directly analyzed and suffice to provide estimates. For an ongoing epidemic, this method could be used to estimate the number of secondary cases infected by a primary case-patient, but only for periods from which all secondary cases would have been detected. For severe acute respiratory syndrome (SARS), the required lag would be on the order of 15 days (95th percentile of the distribution of the generation interval described by Lipsitch et al.) (*3*).

In this report, we show how to estimate the reproduction number in an ongoing epidemic, which will account for yet unobserved secondary cases. The method is applied to data from the 2003 SARS outbreak in Hong Kong (*4*). Using simulated data, we demonstrate how the method may be used for early detection of the effect of control measures.

## Materials and Methods

### Statistical Framework

We propose a Bayesian statistical framework for real-time inference on the temporal pattern of the reproduction number of an epidemic. Here, the reproduction number *R_t_* for day *t* will be defined as the mean number of secondary cases infected by a case with symptom onset at day *t*. Denoting *n_t_* as the number of cases with symptom onset at day *t* and *X_t_* as the number of secondary cases they infected, the reproduction number *R_t_* is the ratio *X_t_*/*n_t_*, defined for *n_t_*>0.

Assume that we would like to compute the daily values *R_t_* from day 0 to present day *T*, before the epidemic has ended. Although daily incident case counts can be known up to day *T*, provided no delay in reporting occurs, the corresponding counts of secondary cases *X_t_* cannot. Secondary case-patients infected before day *T*, whose illness had a long incubation time, may have clinical onset only after day *T*. Furthermore, since the exact chain of transmission is seldom observed in practice, attributing secondary cases to previous cases is difficult. Focusing on these 2 issues, we show that the daily counts of symptom onset available until day *T* are sufficient to estimate *R_t_*.

A 3-step construct is necessary. We first predict the eventual number of late secondary cases (as yet unobserved), for cases reported at day *t*, assuming the number of early secondary cases (reported before day *T*) is known. The method described by Wallinga and Teunis (*2*) is then used to estimate the number of early secondary cases from the daily counts of symptom onsets. These 2 steps are finally combined and yield an estimate of the predictive distribution of *R_t_*. Technical details are given in the Appendix. The estimation procedure depends on 3 assumptions: 1) ascertainment of patients whose symptoms appear before day *T* is complete, 2) transmission events are independent, and 3) the generation interval, the time from symptom onset in a primary case to symptom onset in a secondary case, has a known frequency distribution.

### Data from Hong Kong

The method was retrospectively used to analyze the SARS outbreak in Hong Kong. The data consisted of the dates of symptom onset of the 1,755 case-patients who were detected in Hong Kong in 2003 (*4*).

### Simulated Data

Using simulations, we explored the ability of the method to quickly detect the effect of control measures. Five hundred epidemics were simulated with the following characteristics. During the first 20 days of the epidemics, the theoretical reproduction number was 3. Control measures were implemented at day 20. In a first scenario, control measures were completely effective (no transmission occurred after day 20). In a second scenario, the theoretical reproduction number after control measures were implemented was 0.7. Details on the simulations are available from the corresponding author.

In a simulation study, the bias and precision of the real-time estimator were investigated in situations in which the theoretical reproduction number remained constant with time. We also evaluated the effect of the length of the generation interval on the results. Detailed information can be obtained from the corresponding author.

## Results

### Application to Hong Kong SARS Data

**Figure 1:**
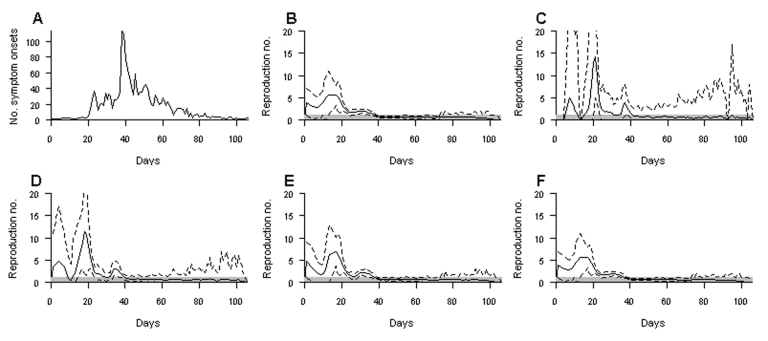
Application of real-time estimation to the severe acute respiratory syndrome outbreak in Hong Kong. A) Data. B–F) Expectation (solid lines) and 95% credible intervals (dashed lines) of the real-time estimator of *R_t_* were calculated at the end of the epidemic (B) and after a lag of 2 (C), 5 (D), 10 (E), and 20 (F) days. The gray zones indicate that *R* is <1.

Figure 1A shows the dates of symptom onset of the 1,755 SARS patients detected in Hong Kong in 2003. Figure 1B–F shows the expectation and 95% credible intervals of the predictive distribution of *R_t_* based on data available at the end of the epidemic and after a lag of 2, 5, 10, and 20 days.

After a lag of 2 days, the 95% credible intervals were wide and displayed an undesirable feature: they sharply decreased to 0 as soon as no cases had been observed for 2 consecutive days (Figure 1C; note especially days 1–4 and 13). After a 5-day lag, this undesirable feature had vanished (Figure 1D).

With lags >5 days, the trends of expected values were relatively similar, with a peak around day 20, a decreasing trend after this date, and the expectation of *R_t_* decreasing to <1 around day 40. These observations suggest that after a lag of only 5 days, the temporal trends in the expectation of *R_t_* are well captured. For a lag of 5 days, the credible interval of *R_t_* was wide when <20 cases were detected (periods 0<*t*<20 and *t*>63), but was relatively narrow when more cases were detected (period 21<*t*<62). As expected, the width of the credible interval narrowed as the lag increased and more complete data were available. The expectations and credible intervals were very similar for lags of 10 and 20 days, 67.8th and 99.7th percentiles, respectively, of the distribution of the SARS generation interval described by Lipsitch et al. (*3*). No difference was detected between retrospective and 20-day estimates.

### Detecting the Effect of Control Measures

**Figure 2:**
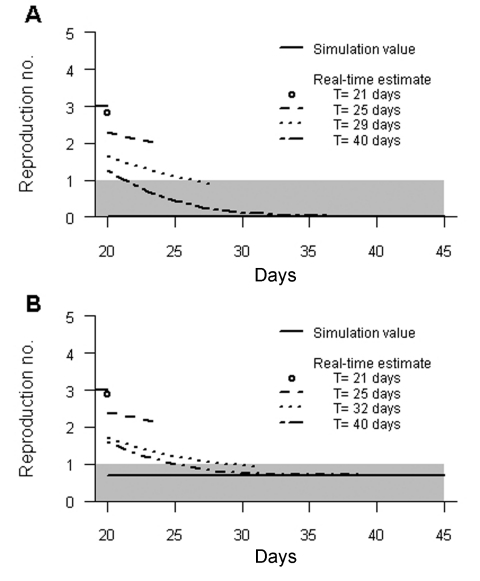
Average expectation of the temporal pattern of *R_t_* after implementation of control measures according to the day *T* of the last observation. A) Completely effective control measures. B) Limited control measures. Simulation values of *R* are also given: before day 20, *R* = 3; after day 20 *R* = 0 (A) and *R* = 0.7 (B). The gray zone indicates that *R* is <1. Information that the average expectation of *R* has passed <1 was obtained 9 (A) and 12 (B) days after control measures were implemented.

In Figure 2, the method is used to estimate the impact of control measures implemented on day 20 in the simulated datasets with completely effective or limited control measures. The curves show the temporal pattern of *R_t_* based on an average over the 500 simulated datasets as a function of *T*. Even when control measures are completely effective, based on data available up to day 21, the average expectation of *R_20_* is ≈3. Based on data available up to day 25, a downward trend is apparent, whereas based on data available up to day 29, the average expectation of *R_t_* is <1 from *t* = 27 days. Based on data available up to day 40 (20 days after the implementation of the control measures), the estimates indicate that the threshold value 1 is crossed at day 22, which is 2 days after control measures were implemented. With limited control measures, the observed changes are qualitatively the same, although slightly more time is required for *R_t_* estimates to decrease to <1.

## Discussion

Our statistical framework provided real-time estimates of the reproduction number of an epidemic, and thus quickly showed the impact of control measures. In simulations of SARS-like diseases, the derived estimator detected the decrease of *R_t_* only 5 days after control measures were implemented. Furthermore, the average estimate had crossed the threshold value of 1 only 9 days after control measures were implemented.

In theory, the method could be applied to communicable diseases with the following characteristics: 1) no asymptomatic cases; 2) no underreporting; 3) knowledge of the generation interval. The list of communicable diseases that could be monitored is therefore relatively large, although it does not include diseases such as influenza, for which the proportion of asymptomatic or unreported cases may be large. In practice, the delay until estimates of the reproduction number become reliable will depend critically on the generation interval distribution. For SARS, when the reproduction number was constant over time, our real-time estimates were almost unbiased after only 1 day. With the original estimator of Wallinga and Teunis (*2*), which is not intended for real-time estimation, downward bias would be a concern for at least 2 weeks after observation. However, real-time estimates obtained for recent days displayed wide 95% credible intervals and zero-width intervals when no cases had been observed or reported for a few days. Here, owing to the relatively short generation interval of SARS (mean 8.4 days) (*3*), reliable estimates were obtained after only 5 days, albeit with wide credible intervals, and they were consolidated after 10 days. These lags corresponded to the 20th and 70th percentiles of the SARS generation interval (*3*). When the generation interval doubled, the time delay required to detect the effect of control measures implementation or to consolidate estimates roughly doubled.

We assumed that the distribution of the generation interval was known and remained unchanged during the course of the outbreak. In practice, however, this distribution is derived from a subset of traced cases. If the subset is small, e.g., the case at the beginning of an emerging disease outbreak, uncertainty will be large. Furthermore, the generation interval may decrease during the course of the outbreak because of quicker interventions, leading to possible bias in the estimates of *R* (*2*). Further developments of the method could take these issues into consideration. For example, one could use information on traced cases as it accrues to sequentially estimate the generation interval. Depending on how cases are traced during the epidemic, changes in the generation interval could also be monitored.

The approach smoothed the temporal pattern of the reproduction number, leading to overestimation of *R* in the week after control measures were implemented. We are trying to find a correction factor for this bias in ongoing research.

The method has a natural real-time implementation in which 1) a first estimate of the reproduction number is available after a lag that depends on the generation interval, and 2) while the epidemic goes on, the estimate is consolidated, and its credible interval narrows. Incorporation of such a statistical estimation framework into real-time surveillance of future infectious disease outbreaks would enhance the ability of epidemiologists to provide timely advice to public health policymakers.

## Appendix

### Statistical Framework

Denoting *n_t_* the number of cases with onset at day *t* and *X_t_* the number of cases they infected, the reproduction number *R_t_* is simply the ratio *X_t_ /n_t_* defined for *n_t_*>0. Here, we define a method to obtain the predictive distribution of *R_t_* given the available data at day *T*, where data *I(T) = {n_t_}_0< t <T_* are the daily counts of incident onsets, assuming that the density *w(.)* of the generation interval is known. We will make use of the decomposition *X_t_* = *X_t_^-^(T)* + *X_t_^+^(T)*, where the number of secondary cases *X_t_* from cases with onset at day *t* has been split in those with onset before *T* (*X_t_^-^(T)*) (early secondary cases), and those with onset after *T* (*X_t_^+^(T)*) (late secondary cases).

The construction of a global estimator is carried out in 3 stages. First, we consider the problem of right censoring, under the assumption that the exact chain of transmission has been observed until day *T*. In this situation, *X_t_^-^(T)* is observed while *X_t_^+^(T)* is censored and must be predicted, conditional on *X_t_^-^(T)* and *n_t_*, to allow computation of the predictive distribution of *R_t_*. Second, when the exact chain of transmission has not been observed, the number of early secondary cases, *X_t_^-^(T)*, is not available. Following the recommendations of Wallinga and Teunis (*1*), we show that it is possible to compute the distribution of *X_t_^-^(T)* given *I(T)*. Finally, the conditional distributions of *X_t_^+^(T)* given *X_t_^-^(T)*, *n_t_* and *X_t_^-^(T)* given *I(T)* are combined to derive the distribution of the reproduction number *R_t_* conditional on *I(T)*. All distributions presented are conditional to the number *n_t_* of symptom onsets at day *t*, but notation is omitted for the sake of clarity.

### Distribution of X_t_^+^(T) | X_t_^-^(T)

We assume that *X_t_* is Poisson distributed with mean *n_t_ λ_t_* and choose a vague gamma *prior* distribution for λ*_t_* with shape parameter α = 10^-5^ and rate β = 10^-5^.

Conditional on *X_t_*, the number *X_t_^-^(T)* of early secondary cases is binomial with parameters *X_t_*, *W_tT_*, where *W_tT_* is the probability that the generation interval is <*T – t*. It follows that *X_t_^-^(T)* | λ*_t_* is Poisson distributed with mean *n_t_ λ_t_ W_tT_*. The same argument would show that *X_t_^+^(T)* | λ*_t_* is Poisson with mean *n_t_ λ_t_ (1 – W_tT_)*. Given λ*_t_*, *X_t_^+^(T)* and *X_t_^-^(T)* are independent so that
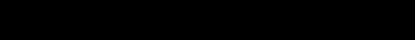
With Bayes' theorem,
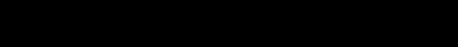
where
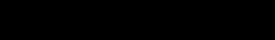
Eventually, we obtain that the distribution *X_t_^+^(T)* | *X_t_^-^(T)* = *y* is negative binomial with parameters *p = (n_t_W_tT_ + β)/(n_t_ + β*), *m* = *y + α* and probability



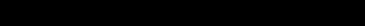



### Distribution of X_t_^-^(T) | I(T)

In practice, the exact realization of *X_t_^-^(T)* is unknown, and inference must be based on *I(T)* alone. Wallinga and Teunis (*5*) have shown that the probability that a case detected at day *k*<*T* has been infected by a case detected at day *t*<*T* is
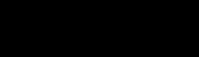
where *1{.}* is the indicator function. The distribution of *X_t_^-^(T)* given *I(T)* is a sum of independent binomial distributions

*X_t_^-^(T)* | *I(T)* ~ ∑*_k <T_* Bin(*n_k_*,*p_tk_*)

This probability distribution may be determined numerically.

### Distribution of R_t_ | I(T)

Using the decomposition in early and late secondary cases, we obtain
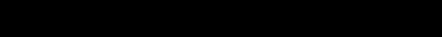
After calculation, we find that the expectation and variance of *X_t_* | *I(T)* are functions of the expectation and variance of *X_t_^-^(T)* | *I(T)* alone, derived with the method of Wallinga and Teunis:
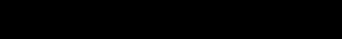


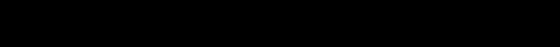
For the vague *prior* we specified for λ*_t_*, we obtain



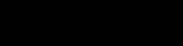





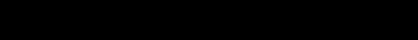



As expected, the average proportion of secondary cases detected before *T* is *W_tT_*. The first term of the variance is related to our imperfect knowledge of the realization of *X_t_^-^(T)* while the second term is related to the natural randomness of *X_t_^+^(T)*. We stress that when the lag between day *t* and day *T* is large (i.e., *W_tT_* ≈1), our estimates are similar to those of Wallinga and Teunis for complete epidemics.

Given *X_t_* | *I(T)*, the derivation of the predictive distribution of the reproduction number *R_t_* is straightforward considering the deterministic relation *R_t_* = *X_t_*/*n_t_*.
